# Extended, virtual and augmented reality in thoracic surgery: a systematic review

**DOI:** 10.1093/icvts/ivab241

**Published:** 2021-09-20

**Authors:** Arian Arjomandi Rad, Robert Vardanyan, Santhosh G Thavarajasingam, Alina Zubarevich, Jef Van den Eynde, Michel Pompeu B O Sá, Konstantin Zhigalov, Peyman Sardiari Nia, Arjang Ruhparwar, Alexander Weymann

**Affiliations:** Department of Medicine, Faculty of Medicine, Imperial College London, London, UK; Department of Medicine, Faculty of Medicine, Imperial College London, London, UK; Department of Medicine, Faculty of Medicine, Imperial College London, London, UK; Department of Thoracic and Cardiovascular Surgery, West German Heart and Vascular Center Essen, University Hospital of Essen, University Duisburg-Essen, Essen, Germany; Department of Cardiovascular Diseases, University Hospitals Leuven, KU Leuven, Leuven, Belgium; Department of Cardiovascular Surgery, Pronto Socorro Cardiológico de Pernambuco (PROCAPE), Recife, University of Pernambuco, Recife, Brazil; Department of Thoracic and Cardiovascular Surgery, West German Heart and Vascular Center Essen, University Hospital of Essen, University Duisburg-Essen, Essen, Germany; Department of Cardiothoracic Surgery, Maastricht University Medical Center, Maastricht, Netherlands; Department of Thoracic and Cardiovascular Surgery, West German Heart and Vascular Center Essen, University Hospital of Essen, University Duisburg-Essen, Essen, Germany; Department of Thoracic and Cardiovascular Surgery, West German Heart and Vascular Center Essen, University Hospital of Essen, University Duisburg-Essen, Essen, Germany

**Keywords:** Virtual reality, Augmented reality, Extended reality, Thoracic surgery, Surgical simulation

## Abstract

**OBJECTIVES:**

Extended reality (XR), encompassing both virtual reality (VR) and augmented reality, allows the user to interact with a computer-generated environment based on reality. In essence, the immersive nature of VR and augmented reality technology has been warmly welcomed in all aspects of medicine, gradually becoming increasingly feasible to incorporate into everyday practice. In recent years, XR has become increasingly adopted in thoracic surgery, although the extent of its applications is unclear. Here, we aim to review the current applications of XR in thoracic surgery.

**METHODS:**

A systematic database search was conducted of original articles that explored the use of VR and/or augmented reality in thoracic surgery in EMBASE, MEDLINE, Cochrane database and Google Scholar, from inception to December 2020.

**RESULTS:**

Our search yielded 1494 citations, of which 21 studies published from 2007 to 2019 were included in this review. Three main areas were identified: (i) the application of XR in thoracic surgery training; (ii) preoperative planning of thoracic procedures; and (iii) intraoperative assistance. Overall, XR could produce progression along the learning curve, enabling trainees to reach acceptable standards before performing in the operating theatre. Preoperatively, through the generation of 3D-renderings of the thoracic cavity and lung anatomy, VR increases procedural accuracy and surgical confidence through familiarization of the patient’s anatomy. XR-assisted surgery may have therapeutic use particularly for complex cases, where conventional methods would yield inadequate outcomes due to inferior accuracy.

**CONCLUSION:**

XR represents a salient step towards improving thoracic surgical training, as well as enhancing preoperative planning and intraoperative guidance.

**Figure:**
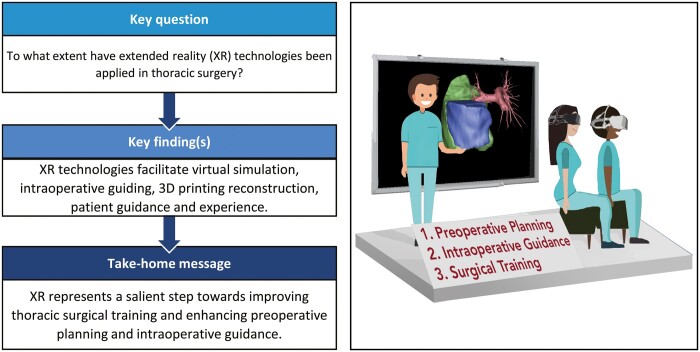

## INTRODUCTION

Extended reality (XR), encompassing both virtual (VR) and augmented reality (AR), allows the user to interact with a computer-generated environment based on reality. VR has exponentially developed over the past four decades to encompass technology whereby users can visualize, explore and interact with various degrees of seemingly real 3D-generated computer environments [1]. In the setting of thoracic surgery, XR tools allow for anatomical assessment, surgical training through lifelike procedural simulations, preoperative planning of surgeries and biopsies, and interoperative guidance [1, 2]. AR furthers this technology by superimposing VR onto real-world environments, displaying information and data on various levels of the reality–virtuality continuum [3, 4].

In essence, the immersive nature of VR and AR technology has been warmly welcomed in all aspects of medicine, gradually becoming increasingly feasible to incorporate into everyday practice. Particularly, since the rapid acceleration of digital medicine development during the COVID pandemic, VR and AR have solidified themselves as ‘only a matter of time’ technologies [5]. Although such XR techniques have been faster utilized in some specialities over others, the technology has not yet matured to similar higher degrees in thoracic surgery. Yet, adoption of XR in this challenging surgical specialty would hold the promise of increasing procedural accuracy, improving surgical decision-making and improving patient safety amongst other benefits [1, 6].

These potential improvements are due, in large part, to specific applications of VR and AR use within thoracic surgery: preoperative virtual simulation of the operation, intraoperative guiding, 3D printing model reconstruction, virtual simulation for training, and patient guidance and experience. Through unmatched visualization and familiarity, VR and AR contribute to the fundamental pillars of surgical practice and mastery.

We aim to evaluate the current applications of XR, VR and AR in thoracic surgery. To our knowledge, no systematic summary of the evidence in this field has been generated.

## METHODS

### Literature search strategy

**Figure 1:**
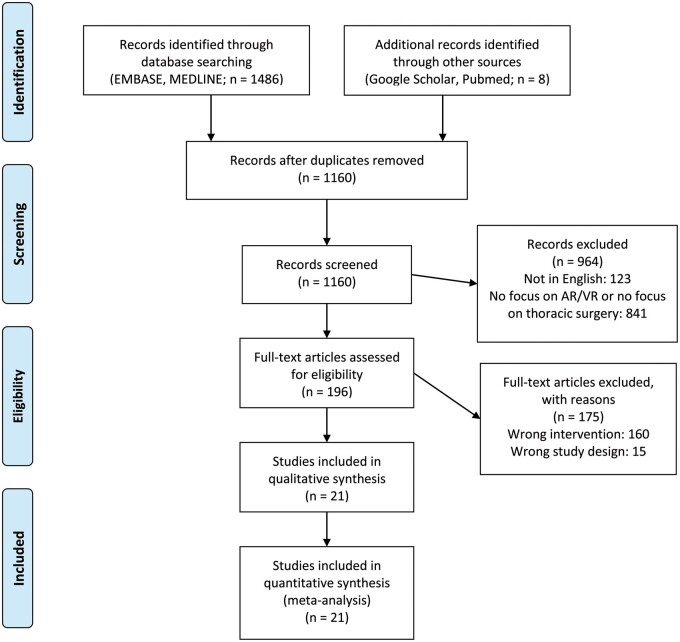
PRISMA flow chart.

A systematic review was conducted in accordance with the Cochrane Collaboration published guidelines and the Preferred Reporting Items for Systematic Reviews and Meta-Analyses (PRISMA) statement. EMBASE, MEDLINE, Cochrane, PubMed and Google Scholar were searched for original articles that explored the use of VR and/or AR in thoracic surgery from inception to December 2020 (Fig. 1)*.* The search terms used were: (Virtual reality OR augmented reality OR virtual simulation OR mixed reality OR extended reality or VR or AR) AND (thoracic surgery OR thoracoscopy OR VATS OR Video assisted thoracoscopic surgery OR cardiothoracic surgery). Further articles were identified through use of the ‘related articles’ function on MEDLINE and a manual search of the references lists of articles found through the original search. The only limits used were English language and the mentioned time frame.

### Study inclusion and exclusion criteria

All original articles were included reporting the use of XR (VR or AR) in thoracic surgery. Studies were excluded from the review if: (i) inconsistencies in the data precluded valid extraction, (ii) the study was performed in an animal model or did not include participants or (iii) the number of participants was low (<10 participants). Case reports, reviews, abstracts from meetings and preclinical studies were excluded. By following the aforementioned criteria, two reviewers (R.V. and S.G.T.) independently selected articles for further assessment following title and abstract review. Disagreements between the two reviewers were resolved by a third independent reviewer (A.AR.). Potentially, eligible studies were then retrieved for full-text assessment.

### Data extraction and critical appraisal of evidence

All full texts of retrieved articles were read and reviewed by two authors (R.V. and S.G.T.) and inclusion or exclusion of studies was decided unanimously. When there was disagreement, a third reviewer (A.AR.) made the final decision. Using a pre-established protocol, the following data were extracted: first author, study type and characteristics, number of patients, population demographics, VR or AR system used and main outcomes. For this review, a data extraction sheet was developed and pilot-tested on 3 randomly selected included studies, whereupon the sheet was refined accordingly. Data extraction was performed by 2 review authors (R.V. and S.G.T.) A third author (A.AR.) validated the correctness of the tabulated data.

## RESULTS

### Study selection and characteristics

The literature search identified 1494 articles. Of these articles, 1160 were screened after deduplication, and 196 were read in full and assessed according to our inclusion and exclusion criteria. Following critical appraisal, a total of 21 studies [2, 7–26] published from 2007 to 2019 and incorporating 1570 patients/participants. Figure 1 illustrated the study selection process. All the studies included were retrospective non-randomized studies, with 8 of them being multicentre (Table 1) [7–10, 12, 16, 17, 21].

**Table 1:** Studies included assessing the use of VR/AR in thoracic surgery training

Study	Year	Study characteristics	Population number	Simulation technique employed	Main reported outcomes
Jensen *et al*.	2014	R, M, P	28	• 2 randomized trainee groups. Group 1 computer-based VR simulator from SimSurgery called SEP, simulated VR nephrectomy. Group 2 black-box training. • After a retention period they performed a thoracoscopic lobectomy on a porcine model.	• No difference between the 2 groups was found in terms of bleeding and anatomical and non-anatomical errors. • The performance of the black box trained group was faster during the test task 26.6 min (SD 6.7 min) vs 32.7 min (SD 7.5 min) with VR.
Jensen *et al*.	2015	NR, M, P	103	• 3 groups: novices *n* = 32, intermediates *n* = 45 and experienced *n* = 26. • A VR VATS simulator was tested with a computer monitor as interface. • A complete endoscopic non-rib-spreading technique was taken based on an anterior view of the hilum.	• The graphics and movements were found to be realistic all participants. • Novice and intermediate participants found the scenario to be highly useful. However, usefulness was rated as low by experienced thoracic surgeons. • No statistically significant difference in terms of metric scores was found between all groups.
Jensen *et al*.	2016	NR, M, P	53	• 3 groups: novices *n* = 17, intermediates *n* = 22 and experienced *n* = 14. • Based on the standardized anterior approach, a virtual reality VATS simulator through a screen monitor as interface was developed. • VR simulators used were LapSim (Surgical Science, Gothenburg, Sweden) to perform a VATS right upper lobectomy.	• They established validity evidence for the VATS simulator. • Significant correlations were found between the simulation metric and level of experience of the participants. • A pass/fail level was defines based on mean scores (±1 standard deviation). All of the novice surgeons and 2 of the experiences surgeons failed to pass the simulation based on the calculated mean.
Gopal *et al*.	2018	NR, NM, P	47	• 1 group of medical students. • EndoVR endoscopy simulator (CAE Healthcare, Montreal, Quebec, Canada), a high-fidelity, haptic feedback simulator was used to perform Bronchoscopy simulation. • Bronchoscopy Skills and Tasks Assessment Tool (BSTAT) was used to assess performance.	• A significant increase in BSTAT score, bronchial anatomy knowledge, bronchial navigational skills was noted through VR simulation in medical students.
Jensen *et al*.	2019	NR, M, P	53	• All participants with no experience of VR simulations performed 2 VR VATS operations. • The VATS lobectomy assessment tool (VATSAT) was used consisting of 8 items especially developed to rate trainees’ VATS lobectomies competencies.	• Validity evidence was provided for a novel assessment tool for evaluating VATS lobectomy competence. • The VATSAT proved to be a specific assessment tool for evaluating VATS lobectomy performance. • The participants VATS lobectomy experience was found to correlate to their score in the simulator.
Whittaker *et al*.	2019	NR, M, P	30	• 3 groups: novices *n* = 16, intermediates *n* = 9 and experienced *n* = 5. • Thoracic robotic lobectomy was simulated using RobotiX Mentor. The system provides step-by-step instructions to robot-assisted right upper lobectomy.	• Realism was rated 3/5 both for the simulator and the module. • The simulator was rate 3.8/5 as acceptable and 3.8/5 as feasible. • Face validity, acceptability and feasibility were ascertained for simulator.
Qin *et al*.	2019	NR, NM, P	32	• 2 groups: novices *n* = 24, experienced *n* = 8. All participants were thoracic surgeons. • The VatsSim-XR simulator consisting of a 3D display, haptic enabled thoracoscopic instruments, endoscope kit and a VR headset. • AR, VR, CVR, MR and black box simulators were compared in peg transfer procedure simulating thoracic tasks.	• Performance level was linked to the experience of the practitioners. • AR provided more balanced training environment based on fidelity and accuracy. • Box and MR have the best realism perception and surgical performance.

AR: augmented reality; M: multicentre; NM: non-multicentre; NP: non-prospective; NR: non-randomized; P: prospective; R: randomized; VATS: video-assisted thoracic surgery; VR: virtual reality.

### Training

A summary of the studies collected and their respective characteristics, simulation technique used and main reported outcomes is provided in Table 1. A total of 7 studies [7–13] were included reporting outcomes on the use of XR, VR and/or AR in thoracic surgery training.

### Preoperative planning

Eleven studies [2, 14–23] were included in the review of XR, VR and/or AR in the preoperative planning of thoracic surgery. Four of these studies, noted with an asterisk, were also included in the review of the intraoperative use of VR and/or AR. A summary of the study characteristics, the simulation technique employed and the reported outcomes are presented in Table 2.

**Table 2:** Studies included in assessing the preoperative use of VR/AR in thoracic surgery

Study	Year	Study characteristics	Population number	Simulation technique employed	Main reported outcomes
Abd-El Gawad *et al.*	2014	NR, NM, NP	21	Children between the ages of 18 months and 7 years with foreign body aspirations presented. Virtual bronchoscopy within multidetector CT (MDCT) was used in detecting the tracheobronchial foreign body inhalation. MDCT findings were compared with results of rigid paediatric bronchoscopy as the gold standard.	MDCT detected the foreign bodies in 17 patients whilst rigid bronchoscopy detected it in 18 patients. Conventional rigid bronchoscopy had 3 false positives. MDCT had 1 case of false positive, 1 case of false negative and 2 cases of true positives. MDCT had a sensitivity of 94.4%, specificity of 75% and accuracy of 90.4%.
Hu *et al.*	2007	NR, NM, NP	17	Participants of varying surgical skills predicted the resectability of lung cancers using 3D and 2D images of 6 anonymous patients. Virtual 3D renderings of the thorax were produced from CT scans and compared with 2D CT images.	3D rendering enhanced the confidence of the prediction by ∼20% as compared to 2D images. 3D rendering increased the accuracy of predicted resectability by ∼20%. It also decreased the planning time by ∼30%. It also reduced the workload by ∼50%, in comparison to 2D CT scans. All participants preferred viewing 3D displays to reading 2D CT images for preoperative planning.
Sato *et al.*	2017	NR, M, P	500	Patients that required sublobar lung resection or had lesions that were anticipated to be difficult to identify intraoperatively were selected. Preoperative virtual-assisted lung mapping (VAL-MAP) to intraoperatively localize pulmonary lesions using 3D images and bronchoscopic dye injections under regular fluoroscopy was used.	Complications occurred in 4 patients (0.8%). Marks were identifiable during operation in ∼90%. The successful resection rate was ∼99%. The contribution of VAL-MAP to surgical success was highly rated by surgeons resecting pure ground-glass nodules (*P* < 0.0001), tumours <5 mm (*P* = 0.0016) and performing complex segmentectomy and wedge resection (*P* = 0.0072).
Sato *et al.*	2018	NR, M, P	153	Patients that required sublobar lung resection or required careful determination of resection margins were selected. They underwent preoperative virtual-assisted lung mapping (VAL-MAP) to allow for intraoperative localisation of pulmonary lesions using 3D images and bronchoscopic dye injections under regular fluoroscopy.	131 wedge resections were performed (71.2%), 51 segmentectomies (27.7%), and 2 other surgical procedures were performed (1.1%). Successful resection was achieved in 178 lesions (87.8%), and VAL-MAP markings successfully aided in the identification of 190 lesions (93.6%). Multivariable analysis showed that the most significant factor affecting resection success was the depth of the necessary resection margin (P = 0.0072).
Sekine et al.	2019	NR, NM, P	58	Preoperative virtual sublobar surgical resection simulations to determine the appropriate tumour resection margin.	The average number of virtual segmentectomies performed was 4.6 ± 1.6. The success rate of transbronchial ICG injections was 89.2% (58/65). The shortest distances to the surgical margin by simulation and by actual measurement were 21.5 ± 11.2 mm and 23.5 ± 8.3 mm (p = 0.190). By propensity score matching, operating time, blood loss, length of hospital stays, and postoperative complications were similar between the ICG injection and control groups.
Shentu *et al.*	2014	NR, NM, NP	74	Virtual puncture using radiotherapy planning simulator combined with methylene blue staining for the localization of small peripheral pulmonary lesions.	The average lesion size was 10.4 ± 3.5 mm and the average distance to the pleural surface was 9.4 ± 4.9 mm. The preoperative localization procedure was successful in 75 of 80 (94%) lesions. The shortest distance between the edges of the stain and lesion was 5.1 ± 3.1 mm. Localization time was 17.4 ± 2.3 min. No complications were observed in all participants.
Ueda *et al.*	2012	NR, NM, NP	10	3D lung model created using CT scan images for simulation of pulmonary lobectomy and segmentectomy to estimate the probability that a lung cancer arising in a segment has a safety anatomical margin for resection.	For 1-cm virtual tumours, the mean chance to accept segmentectomy was 33 ± 15%, for 2-cm tumours it was 24 ± 13% and for 3-cm tumours it was 18 ± 12%.

AR: augmented reality; CT: computed tomography; M: multicentre; NM: non-multicentre; NP: non-prospective; NR: non-randomized; P: prospective; R: randomized; VAL-MAP: virtual-assisted lung mapping; VR: virtual reality.

### Intraoperative assistance

In total, 9 studies [2, 21–26] were included which discussed the intraoperative use of XR, VR and/or AR in a variety of thoracic surgical procedures. The summaries, including methodology and reported outcomes, of these respective studies are presented in Table 3.

**Table 3:** Studies included assessing the intraoperative use of VR/AR in thoracic surgery

Study	Year	Study characteristics	Population number	Simulation technique employed	Main reported outcomes
*Itano *et al.*	2010	NR, NM, P	15	Patients included: suspected or proven lung tumours. 3D-rendered, dynamic virtual PET/CT mediastinoscopic images were reconstructed in the tracheobronchial- and vessel-modes. Then standard mediastinoscopic nodal biopsies were performed; afterwards the clinical benefits of the 3D PET/CT virtual movies over the standard 2D tomographic images were assessed.	The technique enhances understanding of spatial and positional relationship between the FDG-avid nodes and the anatomy of the mediastinum. Offers a more detailed virtual depiction of anatomy leading to improved selection of subsequent operative procedures.
*Akiba *et al.*	2011	NR, M, P	11	Twelve operations in 11 patients who had chemotherapy before pulmonary metastasectomy (lobectomy or segmentectomy): 1 segmentectomy, 10 lobectomies and 1 wedge bronchoplasty upper lobectomy, 10 had VATS. Tailor-made virtual lungs were synthesised using 3D multidetector computed tomography (CT) before operation.	Duration tailor-made virtual lung = approx. 10 min The tailor-made virtual lung enhanced the understanding of the patient’s individual anatomy for VATS. Makes it possible to measure distance and angles among pulmonary arteries, veins and bronchi and examining the locations of vessels and bronchi preoperatively.
Sato *et al.*	2013	NR, NM, NP	41	Patients included: lung tumours. Virtual endobronchial ultrasound for transbronchial needle aspiration: Aquarius Thin Client Viewer (TeraRecon, Inc, Tokyo, Japan) was employed to create 3D virtual bronchoscopy images and a computer-based simulation of EBUS-TBNA with input from thin-slice CT images. Virtual EBUS images and videos were used as reference aids during the EBUS-TBNA procedure.	Virtual EBUS was useful particularly when potential target was outside of the typical mediastinal lymph node. May enhance TBNA procedure performance at difficult and high angles. Offers US confirmation of virtual images and real-time monitoring of operational procedure.
Sato *et al.*	2014	NR, NM, P	30	Patients included: hardly palpable lung tumours. Virtual-assisted lung mapping (VAL-MAP), a bronchoscopic multispot dye-marking technique using virtual images, is used preoperatively to determine reference points. Post-VAL-MAP a 3D reconstruction of the lung is performed using fluoroscopy and CT, which aids before and during the VATS operation.	Duration VAL-MAP: 20–60 min, 55 ± 14 min and 21 ± 6 min for single wedge resection (*n* = 7) and 190 ± 43 and 119 ± 35 min for single segmentectomy (*n* = 20). Of 95 marking attempts, 91 visible during the operation (95.7%), 100% success rate for surgical resections using VAL-MAP. 0 adverse events.
*Sardari Nia *et al.*	2019	NR, NM, P	25	25 patients referred for anatomic pulmonary resections were included. 3D reconstruction of the pulmonary anatomy was constructed by inputting CT scans from a dual-source CT scanner into dedicated rendering software (Fujifilm Synapse Vincent system). An interactive 3D reconstruction with virtual resection was created, in which individual structures could be selected and targeted preoperatively. The reconstruction also aided in intraoperative guiding during 3D VATS.	All patients had complete resections; post-interventional complications were grade ≤2 in 96.2% of patients. The preoperative 3D reconstructions of pulmonary vessels and intraoperative guiding were equal to intraoperative findings in 100% of cases. In 15.4% patients, anatomic variations were revealed upon preoperative 3D reconstructions that were confirmed intraoperatively.
*Sato *et al.*	2019	NR, NM, NP	28	Treatment group: 4 patients with 4 lesions, control group: 3 patients with 5 lesions; afterward trial of electromagnetic navigation bronchoscopy (ENB) VAL-MAP in 19 patients. In treatment group: Planned lung markings on CT images are transferred to an ENB system and a portable radiology workstation intraoperatively to create 3D VAL-MAP images including resection markings. Intraoperatively lung markings are evaluated by a single surgeon, also 3D ENB-VAL-MAP is used to make intraoperative adjustments. In control group, conventional VAL-MAP is used, and markings are also evaluated intraoperatively, but no re-adjustments are made.	No significant difference in the success rate regarding intraoperative navigation between the no-adjustment and adjustment groups (36.3% vs 40.0%, *P* = 0.86). However, looking at the markings placed with no successful navigation, the control group had a significantly lower accuracy grade than the treatment group (2.6 ± 0.5 vs 4.5 ± 0.8). Total time: ENB VAL-MAP = 41 ± 14 min vs VAL-MAP = 43 ± 4.9 min.
Yang *et al.*	2019	NR, NM, P	24	24 patients received the VAL-MAP marking procedure before thoracoscopic segmentectomy. Nineteen of those patients also received preoperative CT-guided percutaneous localization post-VAL-MAP; 15 patients received CT-guided localization with dye and microcoil, and 4 patients received only dye. Virtual bronchoscopy is used for VAL-MAP; after VAL-MAP, a microcoil is placed near the lesion through the CT-guided needle localization; then blue dye is injected to set the marking; at the end, a confirmatory CT scan was performed pre-operation. The contribution of VAL-MAP to the respective surgery is evaluated by the performing surgeon.	Of 101 marking attempts made in all the patients, 71 (70.3%) were identified as contributing to the surgery. No complications occurred after the treatment. After training and video demonstration, the successful total marking rate was 85.7%. Median time from VAL-MAP to CT room was 36 min, VAL-MAP to operating room was 61 min.

AR: augmented reality; CT: computed tomography; M: multicentre; NM: non-multicentre; NP: non-prospective; NR: non-randomized; P: prospective; PET-CT: positron emission tomography–computed tomography; R: randomized; VAL-MAP: virtual-assisted lung mapping; VATS: video-assisted thoracic surgery; VR: virtual reality. * Articles reporting on both the preoperative and the intraoperative use of VR/AR in thoracic surgery.

## DISCUSSION

This systematic review summarizes for the first time the knowledge available on the application of XR (VR and AR) in the field of thoracic surgery. Twenty-one original articles were included and categorized based on their focus into the use of VR/AR in: (i) thoracic surgery training, (ii) preoperative planning of procedures and (iii) intraoperative assistance.

### Training

The application of XR in thoracic surgery training has been demonstrated to have wide scope and great applicability, which is set to increase over the upcoming years thanks to advancement in simulation technologies and their expected reduction in cost. Both VR and AR systems have received considerable attention in the surgical world over the past decades thanks to their ability to offer an educational simulation experiences free from possible ethical and hygienic issues. VR has been demonstrated to produce progression along the learning curve, enabling trainees to reach acceptable standards before performing in the operating theatre [27]. In the UK, the usefulness and desire for simulation to be embedded as part of cardiothoracic surgery national training has been expressed by trainees nationwide [28].

However, in thoracic surgery, the application of this technology is limited to 3 known simulators developed to date, 2 focussing on virtual video-assisted thoracic surgery (VATS) [10, 29] and 1 [12] on virtual robotic VATS. Despite this, the current available technology has been shown to produce acceptable levels of validity, precision, usefulness, acceptability and feasibility. All the studies comparing outcomes in groups with different experiences found the outcomes of virtual VATS to correlate to the level of experience of the operator, with the novice and intermediate experienced surgeons finding virtual VATS simulations the most beneficial for their learning (Table 1). These results are in line with the current literature studying the application of virtual simulation in other surgical specialties, finding junior trainees to be the greatest beneficiaries [30].

The current VR VATS technology developed by Jensen *et al.* [10] in Copenhagen illustrates the feasibility of generating and retaining performance metrics in thoracic surgery, as already also shown in other surgical fields. Junior thoracic surgeons can receive immediate feedback on their surgical performance and keep track of their progress and learning, all independent of the opinion of senior surgeons. Through the development of this technology, senior surgeons will also be able to further hone their skills through tailored metrics.

Moreover, XR could potentially lead towards and aid the future establishment and expansion of robotic thoracic surgical programmes, whereas currently practitioner and institutional experience in the field is not yet mature. Whittaker *et al.* [12] developed and tested the first known VR VATS simulator with realism, face validity, acceptability and feasibility being ascertained.

One of the main limitations still present within the world of VR training remains haptic feedback and the sensation of feeling the instruments, which can for now only be partially replicated to reproduce the surgical context. Two of the studies included compared black-box simulation to VR simulation [7, 13]. Jensen *et al.* [7] in their randomized control trial found black-box trained participants to have significantly faster operative time when performing a VATS operation when compared to VR-trained participants. Nevertheless, with the exponential advancement of VR technology, these limitations could soon be addressed.

### Preoperative planning

**Figure 2:**
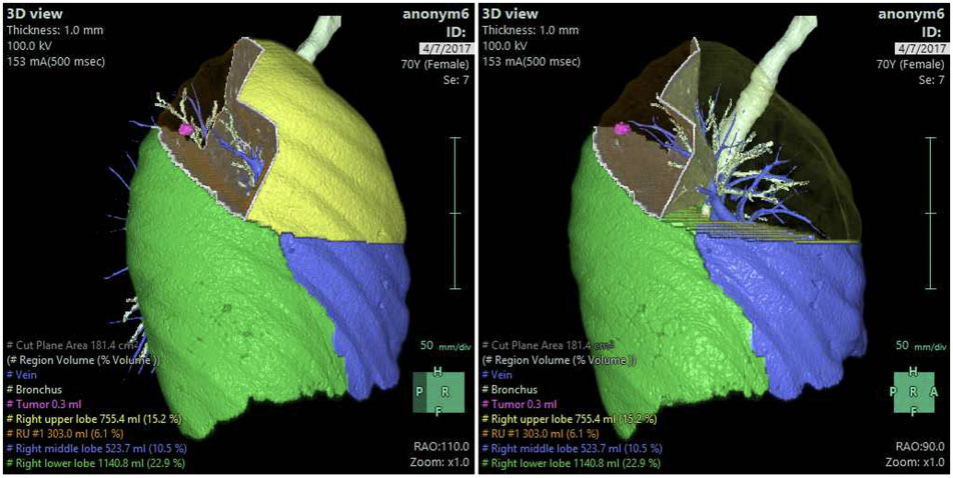
Virtual simulation of segmentectomy.

**Figure 3:**
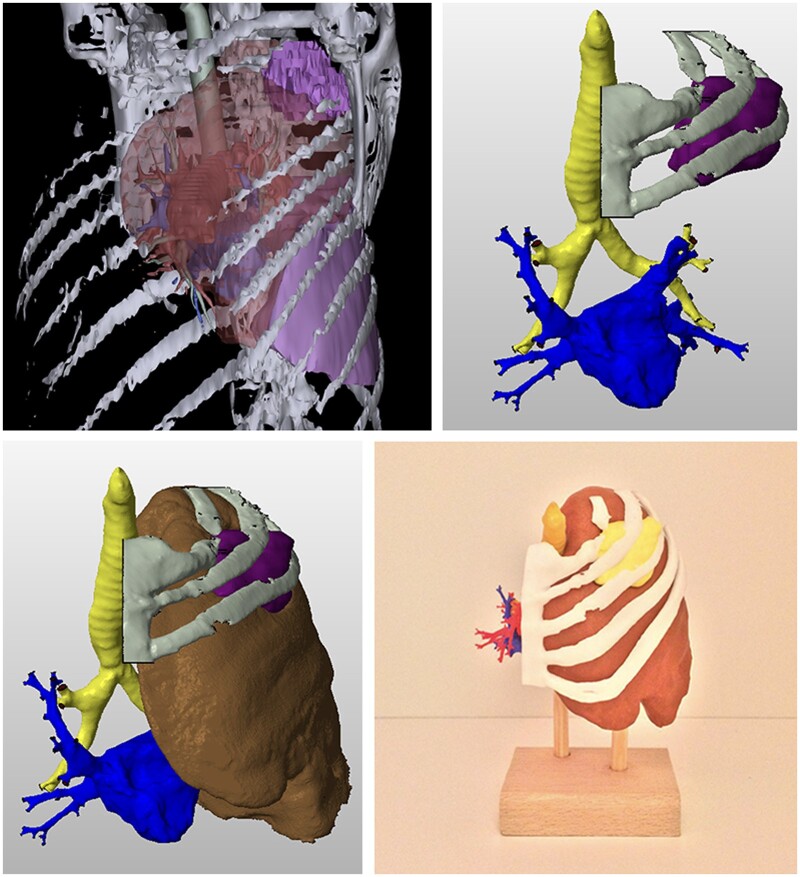
3D reconstruction, processing (STL file) and 3D print of the tumour left upper lobe with thoracic wall invasion.

Preoperatively, XR seems promising in the decision-making and planning of thoracic procedures. Particularly through the generation of 3D-renderings of the thoracic cavity and lung anatomy using CT scans, XR increases procedural accuracy and surgical confidence through familiarization of the patient’s anatomy (Figs 2 and 3). Hu and Malthaner [15] describe the benefit of minimizing the predictability aspect in decision-making and reducing the preoperative planning time and workload of surgeons at various levels when viewing virtual 3D-renderings as opposed to conventional 2D CT scans, and the findings of Itano *et al.* [2] and Akiba *et al.* [21] further support this in the area of nodal biopsies and metastasectomies, respectively. Two studies describe taking the virtual 3D-renderings a step further to initiate simulations prior to procedures. Ueda *et al.* [20] simulated lobectomies and segmentectomies, whereby VR assisted in more accurately estimating the safety anatomical margins for lung cancer resections, and Sardari Nia *et al.* [22] highlighted the benefits of virtual 3D-rendering resection simulations that further aided intraoperative guiding during 3D VATS.

Multi-detector computed tomography (MDCT) virtual bronchoscopy provides another avenue of successfully using VR in preoperative surgical planning by producing a rendering that resembles what can be seen with conventional rigid fibre-optic bronchoscopy. Abd-ElGawad *et al.* [14] describe the potential use of MDCT virtual bronchoscopy in detecting tracheobronchial foreign body inhalations in children with a high sensitivity, specificity and detection accuracy. Whilst MDCT virtual bronchoscopy is yet to enter common clinical practice, it provides the significant advantage over conventional bronchoscopy in its non-invasive nature of detecting foreign bodies. However, it is certainly far from replacing the rigid bronchoscope in detecting obstructions resulting from dynamic airway lesions or subtle mucosal lesions, and it remains an additional tool rather than a potential alternative [31, 32].

Sato *et al.* [17, 23] further developed the use of MDCT virtual bronchoscopy through the creation of a virtual-assisted lung mapping system, combining virtual bronchoscopy and dye-localization techniques. It provides geometric information with multiple markings on the lungs surface to facilitate segmentectomy and wedge resection. Over the past years, Sato *et al.* [16, 17, 23] have described the contribution of virtual-assisted lung mapping in thoracic surgery in numerous multicentre studies in Japan and it looks to be a promising addition to the preoperative, and by extension, the intraoperative arsenal of thoracic surgeons.

### Intraoperative assistance

The application of XR proves to be a promising aid for intraoperative use to treat different lesions in thoracic surgery. In the last 10 years, research regarding the intraoperative use of XR has mainly focused on tumour resection. While postoperative complications were reported upon, to our best knowledge, there has not been any prospective study yet which looks at long-term differences regarding effectiveness, morbidity and mortality between XR-aided and conventional surgery. Hence, this review aims to be a pragmatic tool offering insight into and comparison of different intraoperative implementations of XR-aided thoracic surgery.

The intraoperative use of XR, VR and AR mainly consists of providing comprehensive anatomical mapping and dynamic navigation assistance during tumour resection and nodal biopsies. In total, 9 studies were found which investigated intraoperative use of VR and AR. Of these studies, all studies were in the field of thoracic surgical oncology. Seven of these studies focused on tumour resection, using segmentectomy and lobectomy particularly, and 2 studies focused on lymph node biopsy. No significant complications were reported by any of these studies, which suggest that VR/AR-assisted surgery is safe, which is in line with previous literature [33].

All the studies showed that VR and AR improved intraoperative understanding of the anatomy, which is pivotal for surgical performance. Furthermore, all studies confirmed that VR/AR-aided mapping is at least as accurate than conventional methods of establishing reference markers for surgical navigation. Sato *et al.* [23], being one of the only studies that compared VR/AR-aided surgery to conventional surgery, found that the former was in fact superior, particularly in error-prone situations. In line with this, Sato *et al.* [24, 25] conclude that VR/AR proves to be very beneficial as auxiliary or alternative method for location and resection of hardly palpable lesions, as well as nodules that are lying in difficult planes. These findings suggest that VR/AR-assisted surgery may have therapeutic use particularly for complex cases, where conventional methods would yield inadequate outcomes due to inferior accuracy. However, no study has yet compared the use of VR/AR-aided surgery to conventional surgery for complex cases directly in a blinded setting, hence the findings may be subject to bias.

Itano *et al.* [2], Akiba *et al.* [21] and Sardari Nia *et al.* [22] reported that the intraoperative use of VR/AR, in particular dynamic 3D-virtual lungs constructions, enhances the spatial and positional understanding of the patient’s specific anatomy. Sardari Nia *et al.* [22] additionally pointed out that XR can increase the detection of anatomic variations. These findings further strengthen the position of VR/AR being beneficial intraoperative adjuncts, which may increase the ability of current conventional methods such as VATS and endobronchial ultrasound (EBUS) . That said, most studies used very subjective rating by sole surgeons to evaluate intraoperative use of VR/AR, which undermines the validity of the findings, and hence ultimately complicates drawing conclusions.

### Limitations

This systematic literature review is subject to some limitations. Firstly, most of the studies that were included in our review were single-arm interventional studies, which are known to incur error and bias. Being constrained by the availability of data, the inclusion of more randomized control trials may have increased the validity of the findings of this review. Secondly, most studies included in this review had no blinding methodology, which presents a source of cognitive bias. Additionally, the sample sizes of the studies had a high spread, and often quite small, with all intraoperative studies having <50 patients. In effect, this gives rise to statistical skewing and, therefore, bias.

Finally, a renowned source of bias is publication bias, describing the trend that studies with statistically and clinically significant findings tend to be more likely to be published then their counterpart with no or little significant findings. Thus, this systematic review may be subject to publication bias, enhanced by the fact that case series have been included in this review. Given the detrimental statistical false-positive influence of publication bias, particularly in small to medium samples, no meta-analysis has been conducted.

## CONCLUSION

XR represents a salient step towards improving thoracic surgical training, as well as enhancing preoperative planning and intraoperative performance. Current studies on the use of VR and AR in thoracic surgical training are limited to VATS, but have particularly beneficial for junior trainees, by allowing them to operate in a realistic, yet ethically risk-free setting in order to accelerate their learning process. Moreover, several studies show that VR and AR can reduce preoperative planning time and workload, as well as improving the accuracy of preoperative markings, by allowing tailored models such as virtual 3D-rendering of lungs to be made. Intraoperatively, the use of VR and AR is found to be a safe way to increase intraoperative performance, in complex and error-prone cases, by facilitating deeper spatial understanding of patient-specific anatomy and increasing the detection of anatomic variations. The results of this review are hence promising, but more research particularly using a wider range of thoracic surgical procedures and randomized controlled trial methodology must be conducted in future to confirm and strengthen these findings.

**Conflict of interest:** none declared.

## Author contributions

**Arian Arjomandi Rad:** Conceptualization; Data curation; Formal analysis; Investigation; Methodology; Resources; Software; Validation; Visualization; Writing—original draft; Writing—review & editing. **Robert Vardanyan:** Conceptualization; Data curation; Formal analysis; Investigation; Methodology; Resources; Software; Validation; Visualization; Writing—original draft; Writing—review & editing. **Santhosh G. Thavarajasingam:** Conceptualization; Data curation; Formal analysis; Validation; Writing—original draft. **Alina Zubarevich:** Conceptualization; Data curation; Investigation; Validation; Visualization; Writing—review & editing. **Jef Van den Eynde:** Conceptualization; Data curation; Investigation; Validation; Visualization; Writing—review & editing. **Michel Pompeu B. O. Sá:** Conceptualization; Data curation; Project administration; Validation; Writing—review & editing. **Konstantin Zhigalov:** Conceptualization; Data curation; Project administration; Supervision; Validation; Writing—review & editing. **Peyman Sardari Nia:** Conceptualization; Methodology; Project administration; Resources; Software; Supervision; Validation; Visualization; Writing—review & editing. **Arjang Ruhparwar:** Conceptualization; Project administration; Supervision; Validation; Visualization; Writing—review & editing. **Alexander Weymann:** Conceptualization; Investigation; Methodology; Project administration; Supervision; Validation; Visualization; Writing—review & editing.

## Reviewer information

Interactive CardioVascular and Thoracic Surgery thanks Amit Bhargava, Ilkka Ilonen and the other anonymous reviewers for their contribution to the peer review process of this article.

## ABBREVIATIONS

AR: Augmented reality
MDCT: Multi-detector computed tomography
VATS: Video-assisted thoracic surgery
VR: Virtual reality
XR: Extended reality
